# Soluble Products of *Escherichia coli* Induce Mitochondrial Dysfunction-Related Sperm Membrane Lipid Peroxidation Which Is Prevented by Lactobacilli

**DOI:** 10.1371/journal.pone.0083136

**Published:** 2013-12-16

**Authors:** Arcangelo Barbonetti, Maria Rosaria Caterina Vassallo, Benedetta Cinque, Silvia Filipponi, Paola Mastromarino, Maria Grazia Cifone, Sandro Francavilla, Felice Francavilla

**Affiliations:** 1 Andrology Unit, Department of Life, Health and Environment Sciences, University of L’Aquila, L’Aquila, Italy; 2 Immunopathology Laboratory, Department of Life, Health and Environment Sciences, University of L’Aquila, L’Aquila, Italy; 3 San Raffaele Sulmona Institute, Sulmona, Italy; 4 Institute of Microbiology, University ‘La Sapienza’, Rome, Italy; University Hospital of Münster, Germany

## Abstract

Unidentified soluble factors secreted by *E. coli*, a frequently isolated microorganism in genitourinary infections, have been reported to inhibit mitochondrial membrane potential (ΔΨm), motility and vitality of human spermatozoa. Here we explore the mechanisms involved in the adverse impact of *E. coli* on sperm motility, focusing mainly on sperm mitochondrial function and possible membrane damage induced by mitochondrial-generated reactive oxygen species (ROS). Furthermore, as lactobacilli, which dominate the vaginal ecosystem of healthy women, have been shown to exert anti-oxidant protective effects on spermatozoa, we also evaluated whether soluble products from these microorganisms could protect spermatozoa against the effects of *E. coli*. We assessed motility (by computer-aided semen analysis), ΔΨm (with JC-1 dye by flow cytometry), mitochondrial ROS generation (with MitoSOX red dye by flow cytometry) and membrane lipid-peroxidation (with the fluorophore BODIPY C_11_ by flow cytometry) of sperm suspensions exposed to *E. coli* in the presence and in the absence of a combination of 3 selected strains of lactobacilli (*L. brevis*, *L. salivarius*, *L. plantarum*). A Transwell system was used to avoid direct contact between spermatozoa and microorganisms. Soluble products of *E. coli* induced ΔΨm loss, mitochondrial generation of ROS and membrane lipid-peroxidation, resulting in motility loss. Soluble factors of lactobacilli prevented membrane lipid-peroxidation of *E. coli*-exposed spermatozoa, thus preserving their motility. In conclusion, sperm motility loss by soluble products of *E. coli* reflects a mitochondrial dysfunction-related membrane lipid-peroxidation. Lactobacilli could protect spermatozoa in the presence of vaginal disorders, by preventing ROS-induced membrane damage.

## Introduction

It has been reported that *E. coli*, a frequently isolated microorganism in genitourinary infections [1], may exert adverse effect on human sperm motility [2-4]. Different mechanisms have been proposed concerning this effect. As documented by electron microscopy studies, *E. coli* exhibits the ability to affect spermatozoa directly via cellular interactions mediated by bacterial fimbrias, interacting with receptors in both the sperm tail and head, thus leading to sperm adhesion and agglutination [4-8]. These direct effects of *E. coli* on human sperm motility were found to depend upon the bacterial concentration [4]. 

An adverse effect on sperm motility could be mediated also by leucocytes, which are attracted by *E. coli* to the inflammatory site. Activated macrophages and neutrophils release reactive oxygen species (ROS) and inflammatory cytokines potentially harmful to spermatozoa [9,10]. Accordingly, in *in vitro* models of genital tract infection, the addition of leucocytes in the incubation medium enhanced the harmful activity of *E. coli* on sperm motility [11], by inducing sperm membrane lipid peroxidation [12]. 

It has been recently reported that supernatant obtained from *E. coli* cultures negatively affects mitochondrial membrane potential (ΔΨm), motility and viability of human spermatozoa, even in the absence of white blood cells [13]. Therefore, also soluble factors released by the bacteria appear to be involved in the harmful effect of *E. coli* on spermatozoa.

The inhibitory effect of unidentified soluble products of *E. coli* on sperm mitochondrial function, reported by Schulz et al. [13], does not necessarily account for the loss of sperm motility on a metabolic basis. Evidence has been produced that both in mouse [14] and human spermatozoa [15,16] glycolysis compensates for any lack of ATP production by mitochondria in maintaining sperm motility. However, independently from the impaired mitochondrial ATP generation, a mitochondrial dysfunction could affect sperm motility, when it is accompanied by increased intrinsic mitochondrial generation of ROS. In fact, although low level production of free radicals by spermatozoa plays a positive role in sperm function such as capacitation, acrosome reaction, and sperm hyperactivation [16], high levels of ROS production lead to membrane lipid-peroxidation with subsequent detrimental effect on sperm motility [17,18].

Therefore, the first aim of the present study was to explore the mechanisms by which a possible mitochondrial dysfunction induced by soluble factors of *E. coli* could affect sperm motility, by testing the hypothesis that these factors might trigger a mitochondrial-dysfunction related membrane lipid peroxidation.

Spermatozoa might be exposed to the soluble factors of *E. coli* also in the female lower genital tract, which is an ecological niche where several microorganisms coexist in a dynamic balance. In healthy women, the vaginal ecosystem is dominated by lactobacilli, which are involved in maintaining the normal vaginal microflora by preventing overgrowth of pathogenic and opportunistic microorganisms [19]. The most common vaginal disorder among reproductive age women involving a strong reduction in the number of vaginal lactobacilli is bacterial vaginosis. It has been suggested that the induction of proinflammatory cytokines by an altered vaginal ecosystem may be an unrecognized cause of idiopathic infertility [20]. Intriguingly, lactobacilli could exert anti-oxidant protective effects on spermatozoa. In fact, as we recently demonstrated, soluble factors produced by a mix of 3 selected strains of lactobacilli (*L. brevis* CD2, *L. salivarius* FV2, and *L. plantarum* FV9), which are effective in treating bacterial vaginosis in the form of vaginal tablets [21], prevented lipid peroxidation of sperm membrane induced *in vitro* by ferrous ion, thus preserving sperm motility and vitality [22]. 

The second aim of this study was to evaluate whether lactobacilli could protect human spermatozoa against the harmful effects induced by soluble products of *E. coli*. 

## Materials and Methods

This study was approved by the local Institutional Review Board, ASL n°1 Avezzano-Sulmona-L’Aquila and all subjects signed a written informed consent. 

### Reagents and bacteria

The 5,5′,6′,6′-tetrachloro-1,1′,3,3′-tetraethylbenimidazolyl carbocyanine iodide (JC-1) was purchased from Sigma-Aldrich S.r.l. (Milan, Italy). MitoSOX red and BODIPY (581/591) C_11_ were purchased from Molecular Probes, Inc. (Life Technologies, Monza, MB, Italy). Stock solutions of JC-1, MitoSOX red (MSR) and BODIPY C_11_ in dimethyl sulfoxide (DMSO) were diluted in Biggers, Whitten, and Wittingham (BWW) medium to obtain the working concentrations just before use.

*Escherichia coli* was isolated from vaginal swab of a woman with vaginal symptoms and signs such as discharge and malodour. Microbiological analysis of vaginal discharge demonstrated presence of *Gardnerella vaginalis, Escherichia coli* and *Ureaplasma urealyticum* and absence of lactobacilli. Bacteria were identified according to standard techniques. The mix of active and gamma ray-inactivated lactobacilli (*L. brevis* CD2, *L. salivarius* FV2, and *L. plantarum* FV9) was a kind gift of the VSL Pharmaceuticals (Towson, MD). 

### Semen samples and sperm processing

Ejaculates of 6 normozoospermic healthy donors were collected by masturbation following an abstinence period of 3–7 days. Donors were students or post-graduate students from the University of L’Aquila, who had no known prior male reproductive pathologies including varicocele and infection. All samples were normozoospermic according to World Health Organization (WHO) criteria [23] and did not show leukocytospermia. All samples were left for at least 30 min to liquefy before processing. 

Motile sperm suspensions were obtained by swim-up procedure. Briefly, spermatozoa were washed twice (700 × *g* for 7 min) in antibiotics-free BWW medium. After the second centrifugation, supernatants were removed by aspiration, leaving 0.5 ml on the pellet, and after 30 min of incubation time, supernatants containing highly concentrated motile sperm were carefully aspirated and sperm concentration was adjusted to 5×10^6^/mL. Aliquots from the same semen sample were exposed to different treatments and each experiment was replicated 6 folds using semen from different donors.

### In vitro exposure of motile spermatozoa to bacteria

In order to evaluate the effect of bacteria secretions on spermatozoa, while avoiding direct contact between motile sperms and microorganisms, all incubations were carried out in antibiotics-free BWW medium at room temperature, in air, in a Transwell system (CORNING, New York, NY), where two independent compartments are separated by a 0.4 μm-pore membrane. Motile sperm suspensions (1 mL containing 5×10^6^ spermatozoa) and bacteria (*E. coli* and/or lactobacilli) were placed on the lower and the upper compartment of the chamber, respectively. Lactobacilli were added at concentration of 1×10^8^ CFU/mL. Spermatozoa were exposed to *E. coli* with and without a 30 min-preincubation with lactobacilli mix. After 3 hours incubation, sperm suspensions were recovered for the assessment of sperm motility, vitality, ΔΨm, mitochondrial ROS generation and membrane lipid peroxidation, as described below. 

### Evaluation of sperm motility and vitality

Sperm motility was evaluated by Computer-Aided Semen Analysis (CASA) using ATS20 (JCD, Gauville, France), as previously described [15,17,22]. At least 200 spermatozoa were examined for each sample using standard settings: 30 frames acquired at a frame rate of 60 Hz and a temperature of 37°C in 20-μm deep chambers. Spermatozoa exhibiting an average pathway velocity >5 μm/sec were categorized by the software as motile spermatozoa.

Sperm vitality was evaluated under light microscope by the eosin technique, according to WHO guidelines [23].

### Flow cytometric evaluation of ΔΨm

The fluorescent lipophilic cationic dye JC-1 was used to evaluate the sperm ΔΨm, as previously described [15,17]. This probe possesses the ability to differentially label mitochondria with high and low ΔΨm, by forming multimeric aggregates or monomers, emitting orange-red light or green light, respectively, in the presence of high or low ΔΨm, when excited at 488 nm. After 3-h incubation with microorganisms, donor sperm suspensions, each containing 5×10^6^ spermatozoa, were diluted in 1 mL of phosphate-buffered saline (PBS) to give a final sperm concentration of 1.5-2×10^6^/mL before staining with 0.5 μl of JC-1 stock solution (3 mM in DMSO). Samples were incubated at 37 °C in the dark for 60 min and then analysed using a flow cytometer (Beckman-Coulter Epics XL-4; Beckman Coulter, Inc., Fullerton, CA, USA) equipped with a 15 mW argon-ion laser for excitation. Based on the light scatter characteristics of swim up selected spermatozoa, leucocytes and debris were gated out by establishing a region around the population of interest in the forward scatter/side scatter dot plot on a log scale. For each sample 10000 events were recorded at a flow rate of 200–300 cells/sec. Compensation between FL1 and FL2 was carefully adjusted according to the manufacturer’s instructions. Green fluorescence (480–530 nm) was measured in the FL-1 channel and orange-red fluorescence (580–630 nm) was measured in the FL-2 channel. The percentage of spermatozoa with orange-red fluorescence was evaluated on a 1023 channel scale, using the flow cytometer System II Version 3.0 software (Beckman Coulter, Inc.).

### Flow cytometric assessment of mitochondrial generation of ROS

Mitochondrial generation of ROS, specifically superoxide anion, was evaluated using MitoSOX red (MSR), a lipid soluble cation that is selectively targeted to the mitochondrial matrix and emits red fluorescence when oxidized [17,18]. MSR stock solutions (5 mM in DMSO) were diluted in BWW, added to donor sperm suspensions (20×10^6^/ml) to give a final concentration of 2 μM, and incubated for 15 min at 37 °C. After two centrifugations (600 × *g* for 5 min) in BWW, spermatozoa loaded with the MSR dye were exposed for 3 h to the bacteria in the Transwell system and then analysed by flow cytometry. For each sample 10000 events were recorded at a flow rate of 200–300 cells/sec.

### Flow cytometric assessment of membrane lipid peroxidation

Lipid peroxidation was evaluated using the probe BODIPY 581/591 C_11_, which is incorporated into sperm membranes and responds to free radical attack with a spectral emission shift from red to green [17,22,24]. Albumin-free BWW medium was used because it was found that bovine serum albumin (BSA) binds the lipophilic BODIPY C_11_. BODIPY C_11_ (5 μM) was added to donor sperm suspensions (1.5-2×10^6^/ml), incubated for 30 min at 37°C and washed twice (600 × *g* for 5 min). After 3 h exposure to the bacteria in the Transwell system, sperm suspensions were analysed by flow cytometry. For each sample 10000 events were recorded at a flow rate of 200–300 cells/sec.

### Statistical analysis

Statistical analysis was performed using the R statistical software (version 2.15.2, 2012, The R Foundation for Statistical Computing, Vienna, Austria). The normal distribution of values was assessed with Shapiro–Wilk normality test. Data were analyzed by ANOVA and post hoc comparisons between pairs of groups were performed by the Tukey’s studentized range-honestly significant difference (HSD) test. Correlations were performed by the Spearman correlation test. A multivariate logistic regression analysis of log-transformed values was performed to evaluate the contribution of ΔΨm decrement and lipid peroxidation increment (independent variables) induced by *E. coli* soluble products to the decrement in sperm motility (outcome variable). Statistical significance was accepted when *p*≤0.05. Data were expressed as mean ± SD.

## Results

### Soluble factors of *E. coli* produced a decrease in sperm motility which was prevented by lactobacilli

**Figure 1:**
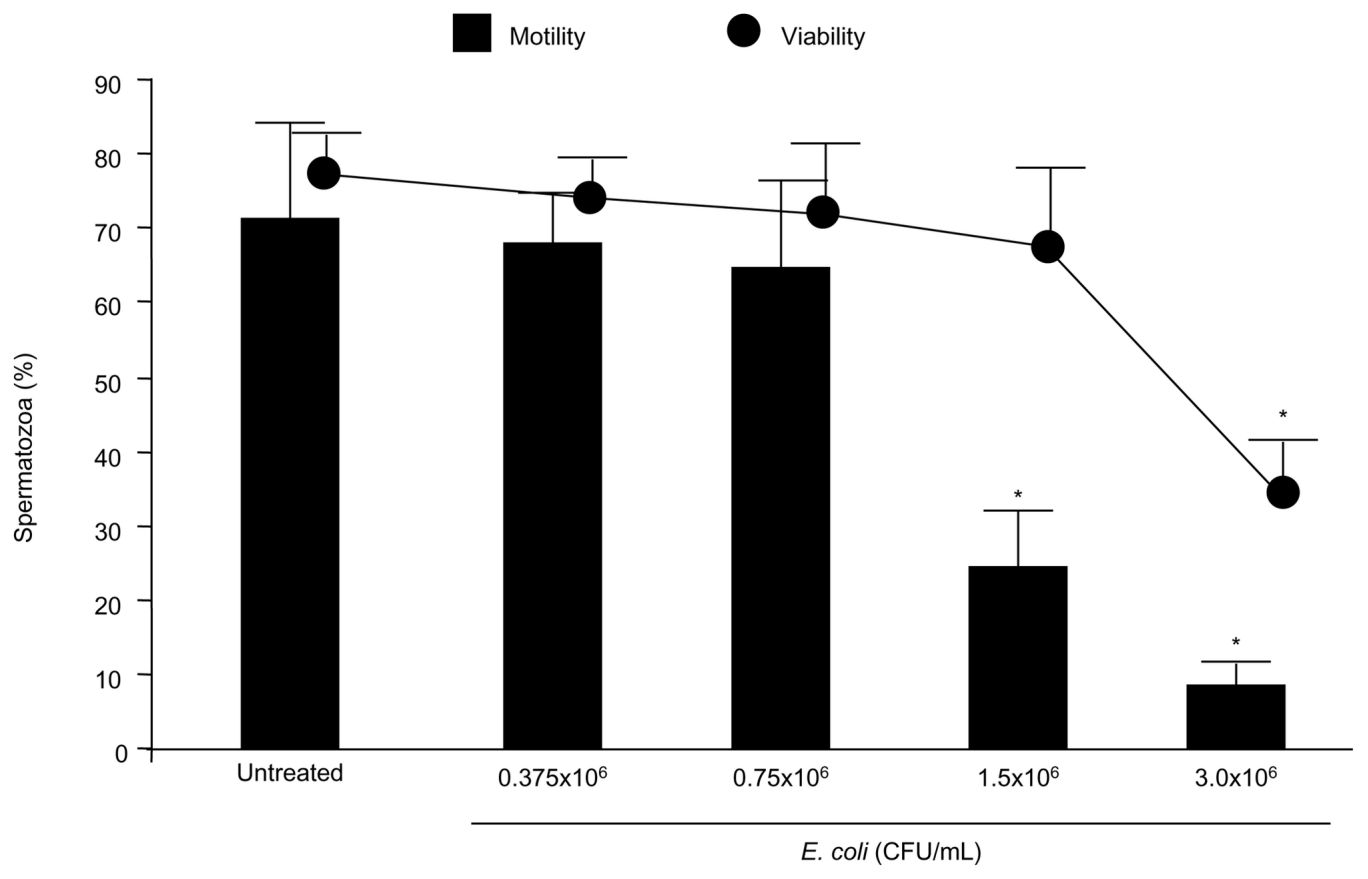
Dose-response effect of *E. coli* on sperm motility and viability. Motile spermatozoa were exposed to *E. coli* for 3 h in a Transwell system, avoiding direct contact between sperm suspension and microorganisms. Mean ± SD of 3 experiments. Overall significance for treatment variations: p<0.001 for both motility and viability; *p<0.05 vs. all the others.

In the first set of experiments, scalar concentrations of *E. coli* were tested in their ability to affect sperm motility and viability. As shown in Figure 1, only *E. coli* concentration ≥1.5×10^6^ CFU/mL affected sperm motility. As higher concentrations of *E. coli* also affected sperm vitality, the effect of 1.5×10^6^ CFU/mL *E. coli* was investigated in subsequent experiments, to rule out non specific mitochondrial dysfunctions due to the cell death.

**Figure 2:**
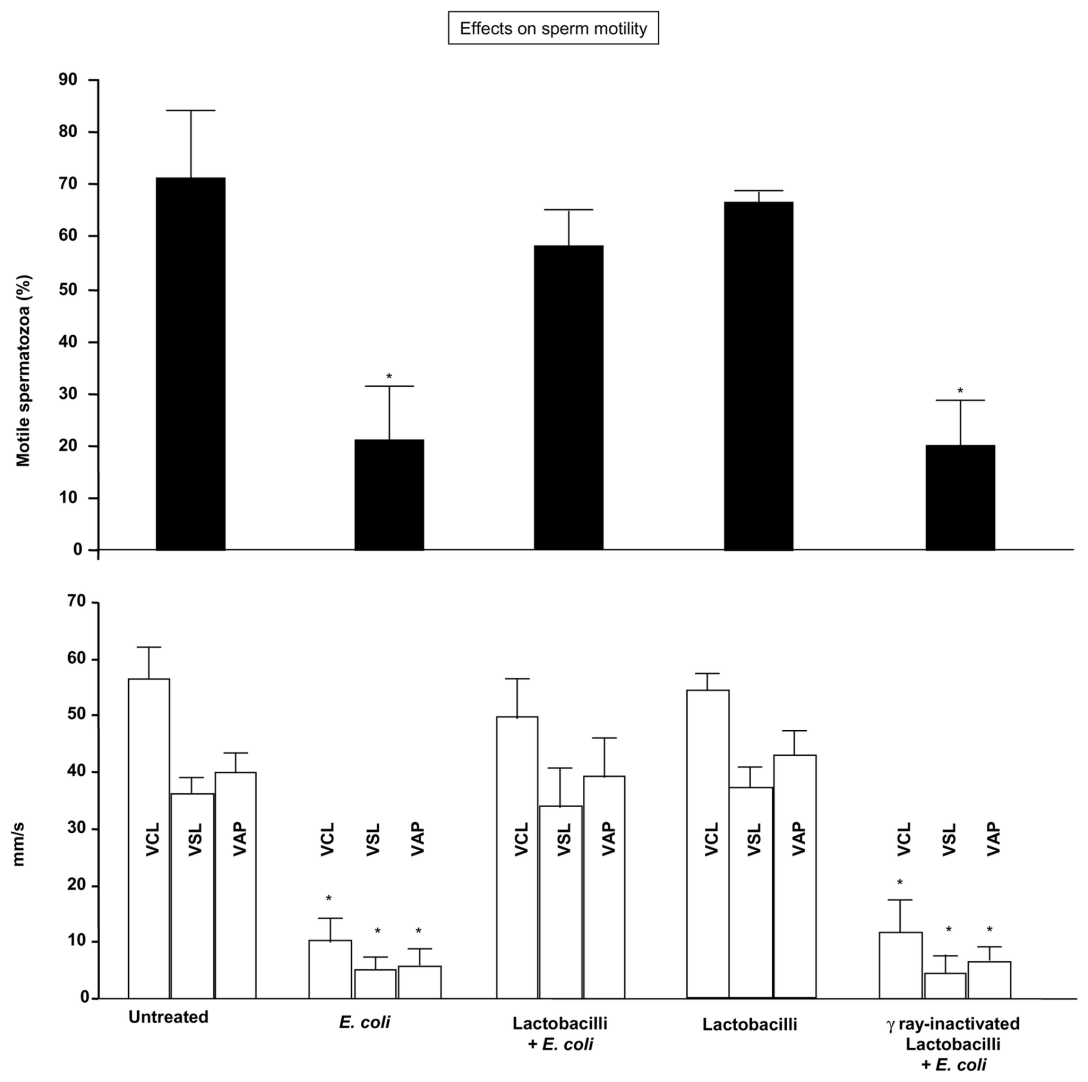
Sperm motility inhibition induced by *E. coli* soluble products and its prevention by lactobacilli. Spermatozoa were exposed for 3 h to *E. coli* (1.5×10^6^ CFU/mL) in a Transwell system with and without a 30 min-preincubation with a mix of *L. brevis* CD2, *L. salivarius* FV2, and *L. plantarum* FV9 (1×10^8^ CFU/mL), before evaluating sperm motility by Computer-Aided Semen Analysis (CASA). No preventive effects were exerted by gamma ray-inactivated lactobacilli. *Top*, effects on the percentage of spermatozoa with average pathway velocity (VAP) >5 μm/sec; *Bottom*, effects on sperm motility quality; VCL, curvilinear velocity; VSL, straight line velocity. Mean ± SD of 6 experiments. Overall significance for treatment variation: *p*<0.0001; *p<0.05 vs. “untreated”, “Lactobacilli + *E. coli*” and “Lactobacilli”.

As shown in Figure 2, the inhibitory effect of *E. coli* on sperm motility was prevented by the addition of the mix of viable active lactobacilli (1×10^8^ CFU/mL). This preventive effect was not observed using gamma ray-inactivated lactobacilli (Figure 2), suggesting a role for soluble factors which are actively secreted by lactobacilli. Incubation with lactobacilli alone did not modify sperm motility compared with the untreated sperm suspensions (Figure 2). The same concentration of lactobacilli (1×10^8^ CFU/mL) was used in the subsequent experiments.

### Soluble factors of *E. coli* induced sperm mitochondrial dysfunction which was not prevented by lactobacilli

To clarify the mechanisms potentially involved in the adverse impact of *E. coli* on sperm motility, as well as in the protective effect of lactobacilli, we focused on mitochondrial function. 

**Figure 3:**
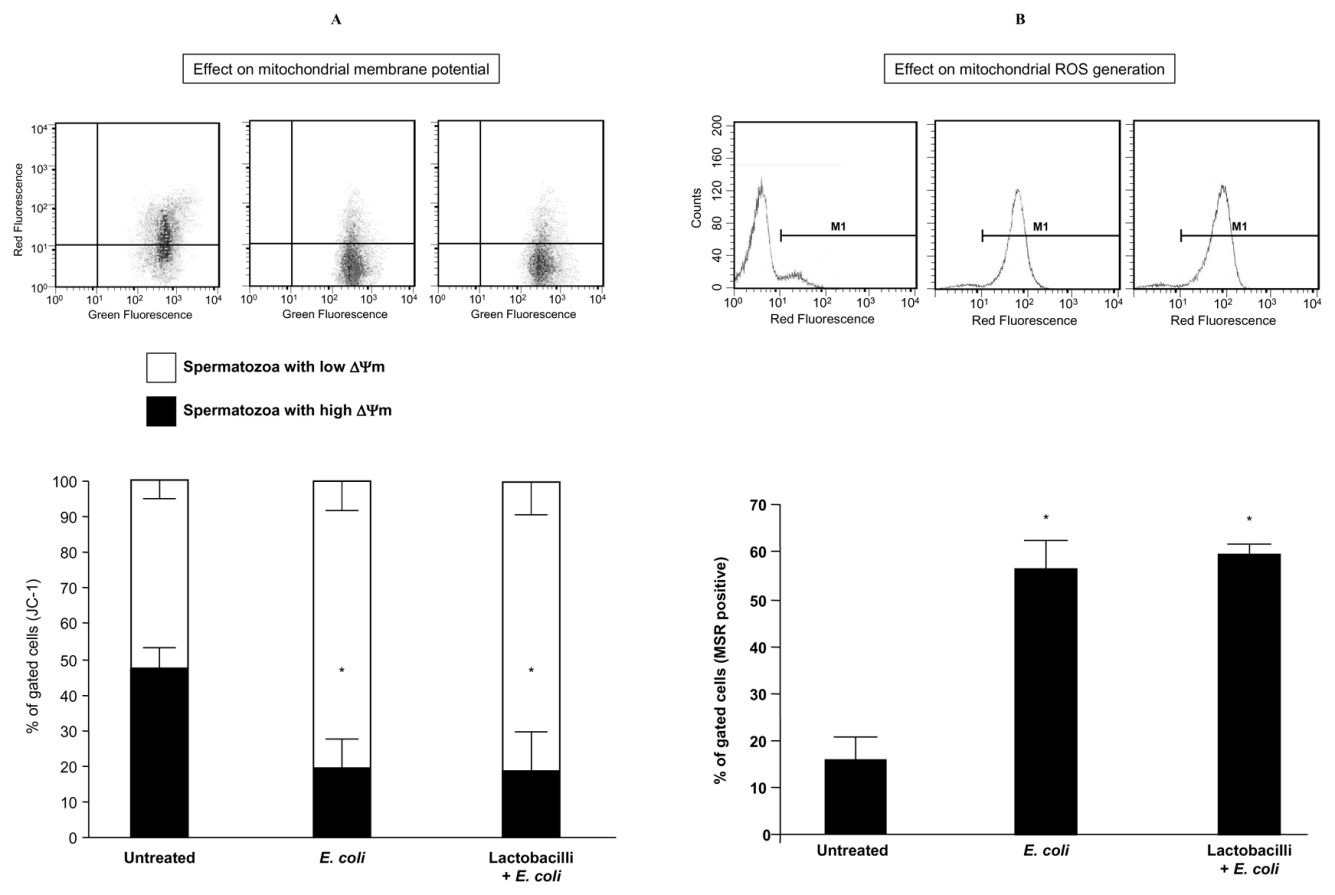
*E. coli* and lactobacilli effects on sperm mitochondrial function. Effect of 3 h exposure in the Transwell system to *E. coli* (1.5×10^6^ CFU/mL) on (**A**) sperm mitochondrial membrane potential (ΔΨm), evaluated with JC-1 and (**B**) mitochondrial generation of reactive oxygen species (ROS), evaluated with MitoSOX red (MSR). Mitochondrial effects of *E. coli* were not prevented by the mix of lactobacilli (1×10^8^ CFU/mL). *Top*, typical flow cytometric dot plot (**A**) and histogram (**B**) of fluorescence; *Bottom*, percentages of spermatozoa with JC-1 and MSR fluorescence. Mean ± SD of 6 experiments. Overall significance for treatment variation: *p*<0.00001; *p<0.05 vs. untreated.

As shown in Figure 3A, sperm suspensions exposed for 3 h to *E. coli* exhibited a significantly lower percentage of spermatozoa with high ΔΨm, with respect to the same sperm suspensions exposed to BWW medium alone. The inhibitory effect of *E. coli* on ΔΨm was not prevented by lactobacilli.

As dysfunctional mitochondria can represent intrinsic sources of free radicals in human spermatozoa [17,18], in subsequent experiments we checked the mitochondrial ROS generation upon 3 h-exposure of sperm suspensions to *E. coli*, in the presence or in the absence of lactobacilli. The exposure to *E. coli* stimulated a significant increase in the percentage of spermatozoa with ROS-generating mitochondria with respect to that observed in control BWW-exposed sperm suspensions and this effect was not prevented by lactobacilli (Figure 3B).

**Figure 4:**
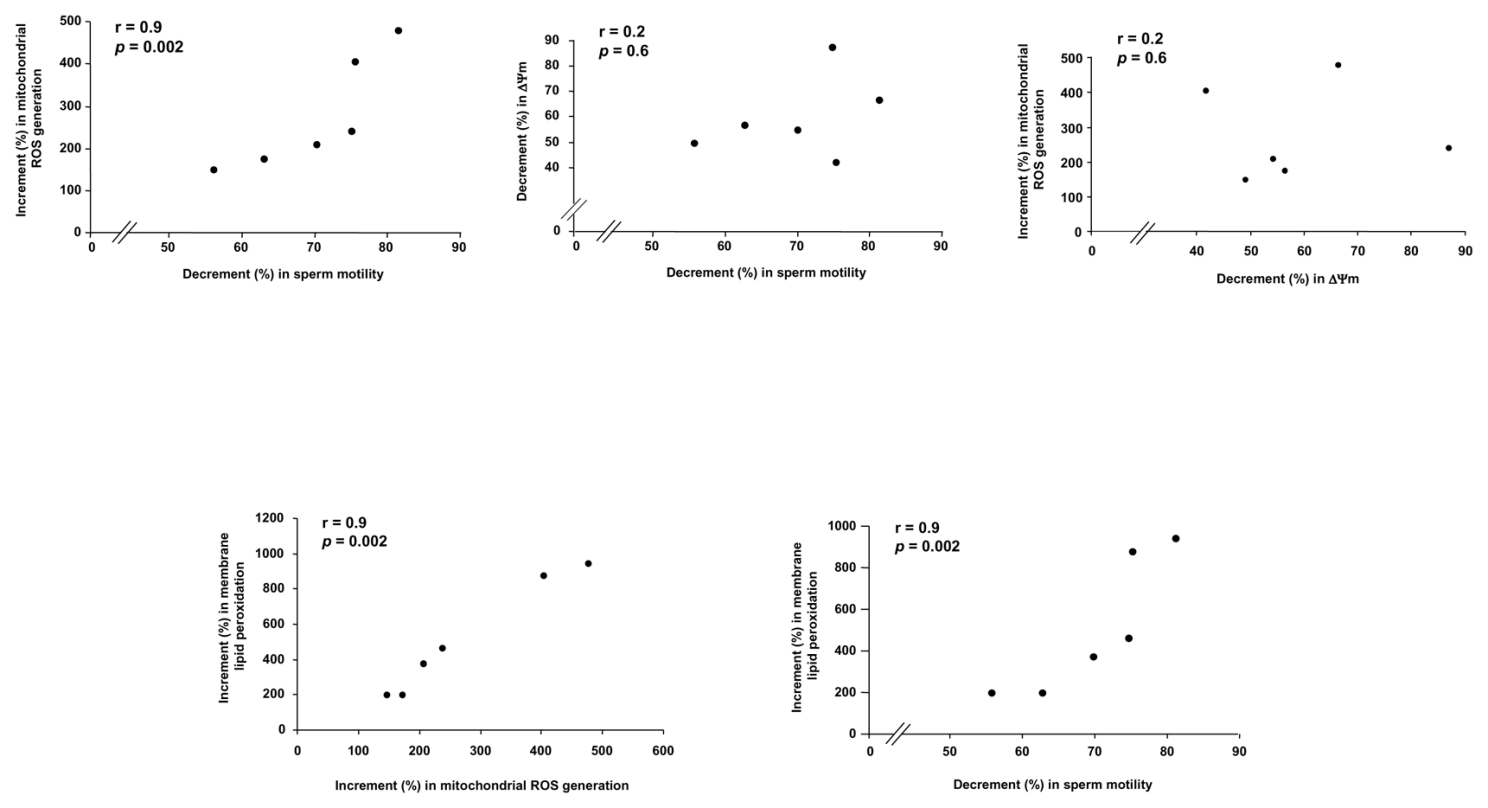
Correlations between *E. coli* effects on spermatozoa. After 3 h exposure to *E. coli* (1.5×10^6^ CFU/mL) in the Transwell system, the increase (%) in sperm mitochondrial generation of reactive oxygen species (ROS) and membrane lipid peroxidation as well as the decrease (%) in mitochondrial membrane potential (ΔΨm) and sperm motility were evaluated with respect to the untreated samples. The percentage of increment/decrement and not crude values were analyzed in order to overcome masking effects due to the inter-donors variability.

The increase in mitochondrial ROS generation induced by *E. coli* soluble factors was highly correlated with the loss of sperm motility, whereas no correlation was found between the decrease in ΔΨm and either the loss of sperm motility or the mitochondrial ROS generation (Figure 4).

### Soluble factors of *E. coli* induced sperm membrane lipid peroxidation which was prevented by lactobacilli

As mitochondrial ROS generation could be responsible for lipid peroxidation of sperm membrane [17,18], we evaluated whether soluble factors from *E. coli*, which induced ΔΨm inhibition (Figure 3A) and mitochondrial ROS generation (Figure 3B), could also promote sperm lipid peroxidation.

**Figure 5:**
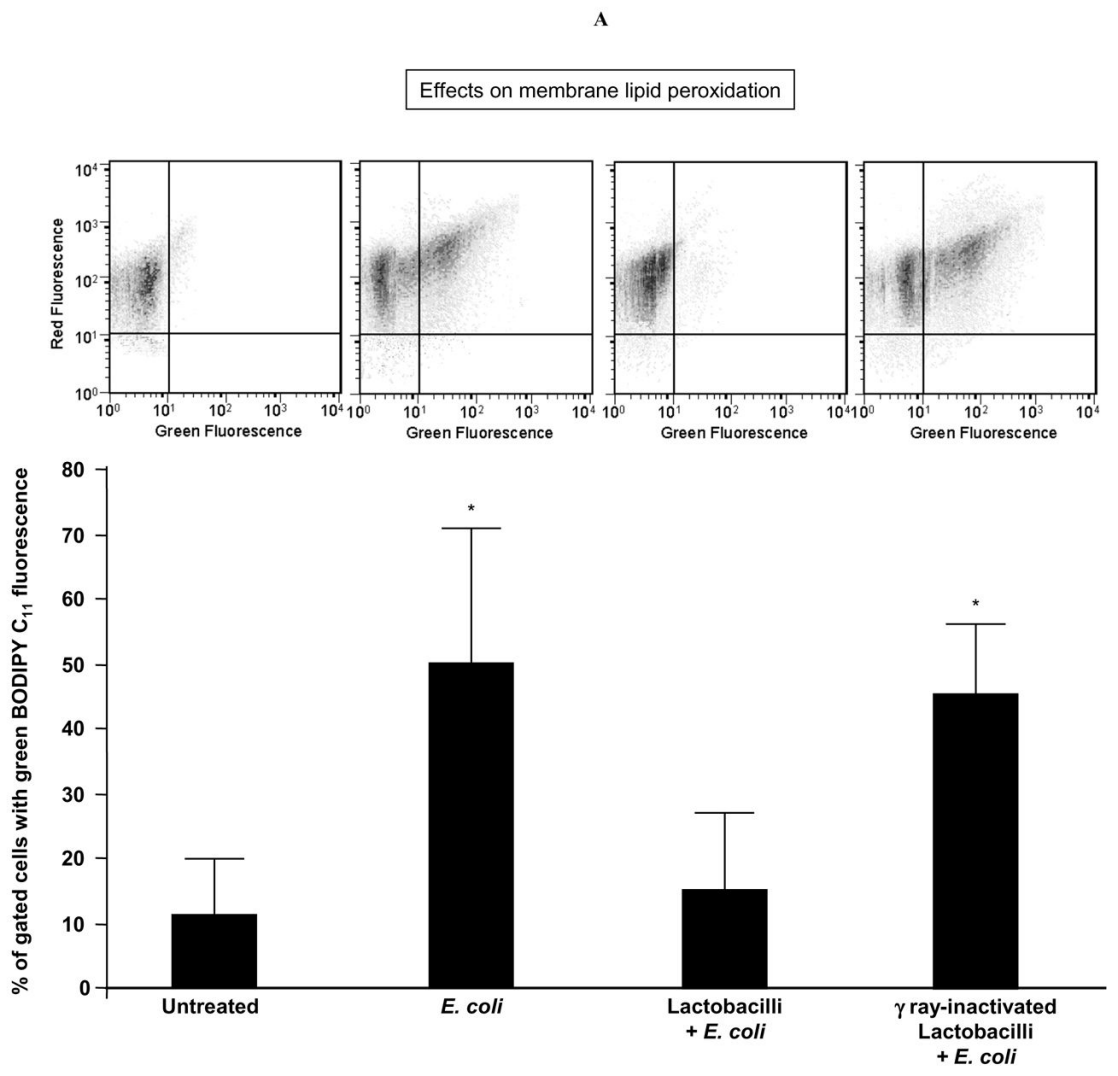
Sperm lipid peroxidation induced by *E. coli* soluble products and its prevention by lactobacilli. Spermatozoa were exposed for 3 h to *E. coli* (1.5×10^6^ CFU/mL) in a Transwell system with and without a 30 min-preincubation with a mix of *L. brevis* CD2, *L. salivarius* FV2, and *L. plantarum* FV9 (1×10^8^ CFU/mL), before evaluating sperm membrane lipid peroxidation with BODIPY C_11_ at flow cytometer. No preventive effects were exerted by gamma ray-inactivated lactobacilli. *Top*, typical double fluorescence dot plots of flow cytometry BODIPY C_11_ analysis. *Bottom*, percentages of spermatozoa with green BODIPY C_11_ fluorescence (indicating membrane lipid peroxidation). Mean ± SD of 6 experiments. Overall significance for treatment variation: *p*=0.00004; *p<0.05 vs. “untreated” and “Lactobacilli + *E. coli*”.

By evaluating the percentages of spermatozoa emitting BODIPY C_11_ green fluorescence, after 3 h-exposure to *E. coli* in the Transwell system, sperm suspensions exhibited a significant increase in membrane lipid peroxidation (Figure 5). Although *E. coli*-induced mitochondrial dysfunction was not prevented by lactobacilli (Figure 3A,B), viable active lactobacilli protected spermatozoa against lipid peroxidation induced by *E. coli* (Figure 5), suggesting the intervention of anti-oxidant factors at the membrane level.

The increase in membrane lipid peroxidation induced by *E. coli* soluble factors was highly correlated with the mitochondrial ROS generation as well as with the loss of sperm motility (Figure 4). At the regression analysis of log-transformed values, the increase in membrane lipid peroxidation significantly explained the decrease in sperm motility (R^2^ = 82%; β coefficient = 0.18; *p*=0.009) whit no additional significant contribution of the decrease in ΔΨm (R^2^ = 10%; β coefficient = 0.16; *p*=0.1).

## Discussion

In keeping with recently reported findings [13], in this study, soluble factors of *E. coli* negatively affected sperm ΔΨm and motility. The effect on sperm motility could not be attributed to simple mechanisms of energy depletion due to ΔΨm inhibition, as this interpretation would ascribe a key role to mitochondrial oxidative phosphorylation in providing energy for sperm motility. Actually, there is growing evidence that flagellar glycolysis compensates for any lack of ATP production by mitochondria in maintaining sperm motility, while mitochondrial gluconeogenesis, supported by pyruvate and lactate, may in turn maintain motility in the absence of extracellular glycolysable substrates [14,15,17,18,25].

The first original datum arising from this study is that the inhibitory effect on sperm motility exerted by soluble factors of *E. coli* was related to an increase in mitochondrial ROS generation and sperm membrane lipid peroxidation. It is well-known that ROS exert detrimental effects on sperm motility, because of lipid peroxidation, affecting membrane integrity and flexibility [9]. Indeed, human spermatozoa are uniquely sensitive to oxidative stress: the high content in polyunsaturated fatty acid of the membrane makes spermatozoa highly susceptible to lipid peroxidation [26]; moreover, spermatozoa are largely devoid of cytoplasm that in somatic cells contains antioxidant enzymes offering a first-line defence against free radicals [9]. 

An adverse effect on sperm motility can be exerted other than by ROS produced by extrinsic sources, also by an excess of ROS production by spermatozoa themselves [9]. Dysfunctional mitochondria represent the major intrinsic source of excessive ROS generation in human spermatozoa [18], able to induce membrane lipid peroxidation and motility loss [17,18,27-29]. The loss of sperm motility induced by soluble factors of *E. coli* was highly correlated with the increase in mitochondrial ROS generation and in membrane lipid peroxidation, but not with the decrease in ΔΨm. At the regression analysis the increase in membrane lipid peroxidation significantly explained the decrease in sperm motility, whereas no additional significant contribution of the decrease in ΔΨm was revealed. Furthermore, no correlation was found between the increase in mitochondrial ROS generation and the decrease in ΔΨm. In keeping with these findings, recent reports demonstrated that in human spermatozoa the mitochondrial dysfunction-related intrinsic ROS generation does not necessarily reflect a decrease in ΔΨm [28,29]. On the whole, the present data indicate that lipid peroxidation is the key determinant of the mitochondrial dysfunction-related motility loss, in line with other experimental [18] and clinical [17] human models. 

A contribution of extrinsic ROS, directly released by *E. coli* is unlikely, as *E. coli* contains antioxidant enzymes and does not release ROS which are generated in its cytosol [30]. Accordingly, only the use of mutants that lack these enzymes allows to demonstrate the *E. coli* ability to generate ROS [31,32]. 

The reasons for the vitality loss induced by very high concentrations of *E. coli* have not been investigated in the present study. The disruption of ΔΨm might also reflect the activation of mitochondrial permeability transition pores with subsequent cytochrome *c* release, a pro-oxidative/pro-apoptotic event, followed by the activation of caspase-9 and caspase-3 and subsequent cell death [33]. A role in exerting apoptotic effects has been also ascribed to lipopolysaccharide, the endotoxin produced by both gram-negative bacteria and *Chlamydia trachomatis* [34,35]. Other *E. coli* toxins, such as α-hemolysin [36] and Shiga-like [37] could also contribute to this effect.

Another major outcome of this study is that the *E. coli*-induced sperm lipid peroxidation was prevented by soluble products of a combination of *L. brevis* CD2, *L. salivarius* FV2, and *L. plantarum* FV9, which also preserved sperm motility. The lack of preventive effect by using gamma ray-inactivated lactobacilli, suggested a role for soluble factors actively secreted by lactobacilli. Furthermore, as lactobacilli did not prevent the *E. coli*-induced mitochondrial dysfunction, their protective effect against membrane lipid peroxidation could reflect the intervention of anti-oxidant factors at the membrane level. Indeed, lactobacilli exhibit antioxidant properties, which provide their protection against ROS attack when they colonize the host mucosal surfaces [38-40]. Accordingly, we have recently reported that the same mix of three selected strains of lactobacilli, used in the present study, at the same CFU concentration, also prevented sperm lipid peroxidation induced *in vitro* by a ferrous ion promoter, thus preserving sperm motility and viability [22]. Intriguingly, it has been recently demonstrated that *L. plantarum* produces carotenoids [40], exerting well known antioxidant actions within biological membranes [41].

In conclusion, this study sheds light on biological mechanisms of the sperm damage induced by soluble products of *E. coli*, that is, mitochondrial dysfunction-related membrane lipid peroxidation and suggests the potential of vaginal lactobacilli in improving the fertilisation potential of the female host, also protecting spermatozoa in the presence of vaginal disorders.
